# Comprehensive mutational analysis of background mucosa in patients with Lugol‐voiding lesions

**DOI:** 10.1002/cam4.3905

**Published:** 2021-05-02

**Authors:** Naoki Akizue, Kenichiro Okimoto, Makoto Arai, Yosuke Hirotsu, Kenji Amemiya, Hirotaka Oura, Tatsuya Kaneko, Mamoru Tokunaga, Kentaro Ishikawa, Yuki Ohta, Takashi Taida, Keiko Saito, Daisuke Maruoka, Tomoaki Matsumura, Tomoo Nakagawa, Motoi Nishimura, Tetsuhiro Chiba, Kazuyuki Matsushita, Hitoshi Mochizuki, Osamu Yokosuka, Masao Omata, Naoya Kato

**Affiliations:** ^1^ Department of Gastroenterology Graduate School of Medicine Chiba University Chiba Japan; ^2^ Genome Analysis Center Yamanashi Prefectural Central Hospital Yamanashi Japan; ^3^ Division of Clinical Genetics and Proteomics Department of Laboratory Medicine Chiba University Hospital Chiba Japan; ^4^ Department of Gastroenterology Japan Community Health care Organization Funabashi Central Hospital Chiba Japan; ^5^ The University of Tokyo Tokyo Japan

**Keywords:** background mucosa, esophageal squamous cell carcinoma, Lugol‐voiding lesions, *NOTCHI*, *TP53*

## Abstract

Somatic mutations including the background mucosa in patients with Lugol‐voiding lesions (LVLs) are still not well known. The aim of this study was to evaluate the somatic mutations of the background mucosa in patients with LVLs (Squamous cell carcinoma (SCC), intraepithelial neoplasia (IN), and hyperplasia). Twenty‐five patients with LVLs (9 with SCC, 6 with IN, and 10 with hyperplasia) were included. A targeted sequence was performed for LVLs and background mucosa using an esophageal cancer panel. Each mutation was checked whether it was oncogenic or not concerning OncoKB. In LVLs, *TP53* was the most dominant mutation (80%). Furthermore, 72% of *TP53* mutations was putative drivers. In background mucosa, *NOTCH1* was the most dominant mutation (88%) and *TP53* was the second most dominant mutation (48%). Furthermore, 73% of *TP53* mutations and 8% of *NOTCH1* mutations were putative drivers. Putative driver mutations of *TP53* had significantly higher allele frequency (AF) in SCC than in IN and hyperplasia. Conversely, putative driver mutations of *NOTCH1* did not have a significant accumulation of AF in the progression of carcinogenesis. Furthermore, in SCC, AF of *TP53* mutations was significantly higher in LVLs than in background mucosa, but not in IN and hyperplasia. Regarding *NOTCH1*, a significant difference was not observed between LVLs and background mucosa in each group. The background mucosa in patients with LVLs already had putative driver mutations such as *TP53* and *NOTCH1*. Of these two genes, *TP53* mutation could be the main target gene of carcinogenesis in esophageal SCC.

**Clinical Trials registry:** UMIN000034247.

## Graphical abstract

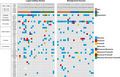

## 1 INTRODUCTION

Esophageal cancer is the eighth leading cause of cancer‐related mortality in the world. Esophageal squamous cell carcinoma (ESCC) is the most common subtype of esophageal cancer, especially in east Asian countries.^1^, ^2^, ^3^ In Japan, ESCC ranks as the 13th most common cancer and the 11th most common cause of cancer‐related death.^4^

Esophageal chronic inflammation caused by carcinogens such as smoking and alcohol induces DNA damage and multiple genetic changes.^5^, ^6^ Therefore, to evaluate the grade of inflammation of the esophagus, Lugol chromoendoscopy with iodine dye is conducted. Past studies have revealed that the severity of esophageal Lugol‐voiding lesions (LVLs) correlates with the risk for the development of ESCC and *TP53* mutation in the background mucosa.^7^, ^8^, ^9^, ^10^ Because the stepwise development of carcinogenesis in ESCC has been suggested, estimation for the carcinogenic potential of LVLs that have no dysplastic change is needed.

In the esophagus, the concept of “field cancerization” is proposed. It means that an accumulation of genetic alterations in the normal mucosa led by repeated exposure to carcinogens induces the development of multiple independent cancers.^11^, ^12^, ^13^ Hence, it could be hypothesized that earlier carcinogenic change occurred in the background mucosa. Hence, evaluating the carcinogenic potential of the normal mucosa is extremely significant.

Recently, the development of next‐generation sequencing (NGS) has revealed gene mutations in several cancers. Subsequently, several mutational analysis data associated with ESCC have been reported from Japan and China.^14^, ^15^, ^16^, ^17^ However, somatic mutations of background mucosa in patients with LVLs are still not clarified. Investigating the genetic changes of the background mucosa could reveal genetic alteration events of early carcinogenesis of ESCC. The aim of this study was to evaluate the details of somatic mutations of background mucosa in patients with LVLs.

## 2 MATERIALS AND METHOD

### 2.1 Study population and sample preparation

We recruited 25 patients with ESCC or a risk of developing ESCC and five healthy controls. The risk of ESCC was defined as (a) post‐ESD (endoscopic submucosal dissection) patients for ESCC, (b) patients with head and neck SCC (HNSCC), (c) patients with chronic esophagitis. Nine patients with ESCC, six patients with intraepithelial neoplasia (IN), and 10 patients with hyperplasia were recruited for this study. Hyperplasia is a condition in which cell proliferation is observed, but there is no obvious abnormality in cell morphology or structure. On the other hand, IN is defined that there are mildly abnormal but not atypical enough to be cancerous. These definitions were made by experts of the Japanese society of pathology. All ESCCs in this study were superficial cancer, eight were stage 0 and one is stage Ⅰ in UICC classification. Healthy controls were defined as without high risk of ESCC and LVLs. When endoscopic resection or endoscopy was performed, tissue was obtained from LVLs (SCC, IN, hyperplasia) and background mucosa defined as iodine‐stained mucosa in each patient because the iodine‐stained mucosa among biopsy sample hardly has neoplastic component.^18^ In the case of healthy controls, tissue was obtained only from background mucosa. All tissues were obtained by the large‐capacity forceps (Boston Scientific, Radial Jaw 4) which can get about 5 mm tissue. The pathological finding of both LVLs and iodine‐stained mucosa from the specimen resected by ESD or EMRC was confirmed. Among the tissue collected by biopsy, for pathological evaluation, additional tissue was obtained from the same LVLs, and the pathological finding was confirmed by hematoxylin‐and‐eosin staining. The tissue collected is shown in Figure S1. All tissue samples were quickly put into Allprotect Tissue Reagent (Qiagen, Hilden, Germany) and preserved at 4°C until deoxyribonucleic acid (DNA) extraction. In addition, peripheral blood was obtained from all 25 patients and five healthy controls before or after endoscopy. Each buffy coat was isolated after centrifugation at 820 × *g* at 4°C for 10°min and stored at −80°C until DNA extraction.

DNA of the tissue sample and the buffy coat of each patient was extracted with a QIAamp DNA Mini Kit (Qiagen, Hilden, Germany). Then, a NanoDrop (Thermo Fisher Scientific, Waltham, MA) was used to measure DNA concentration. Subsequently, extracted DNA was stored at −80°C.

### 2.2 Esophageal cancer (EC) panel

The panel was designed by referring to the previous studies with Ion AmpliSeq designer software (Thermo Fisher Scientific).^19^, ^20^ Seventy significantly mutated genes (SMGs) were included to determine the EC panel in‐house (Table S1). This panel covers SMGs of both ESCC and esophageal adenocarcinoma. The SMGs were selected according to the following criteria: (a) genes often involved in EC, obtained from TCGA and other projects.^14^, ^15^, ^16^, ^17^, ^21^ (b) genes frequently mutated in EC, referring to the COSMIC database (http://cancer.sanger.ac.uk/cancergenome/projects/cosmic). Finally, the panel contained 4410 primer pairs.

### 2.3 Targeted NGS

Extracted DNA was amplified by multiplex PCR with the premixed EC panel and HiFi Master Mix (Ion AmpliSeq Library Kit 2.0). After multiplex PCR, amplicons were treated with FuPa reagent (Ion AmpliSeq Library Kit 2.0) to partially digest the primer sequences and phosphorylate the amplicons for optimization of the sequencing performance. Then, the amplicons were ligated to adapters with barcodes using an Ion Xpress Barcode Adapters kit. The ligated library was purified using Agencourt AMPure XP reagents (Beckman Coulter, Brea, CA), and the library concentration was quantified by real‐time PCR with an Ion Library Quantitation Kit (Thermo Fisher Scientific). Then, the concentration of each library was adjusted to 10 picomolar. The adjusted library was enriched using emulsion PCR with Hi‐Q™ View OT2 Kit (Thermo Fisher Scientific). After loading on an Ion 318 Chip, sequencing was carried out on an Ion PGM (Thermo Fisher Scientific). All procedures were performed following the manufacturer's recommendations. Each Library Kit was the product of Thermo Fisher Scientific.

### 2.4 Data analysis

Sequence data were processed with Ion Torrent Suite Software. Raw signal data were analyzed using Torrent Suite version 5.10 after signal processing, base‐calling adapter trimming, quality score assignment, PCR duplicate removal, read alignment to the reference human genome 19, quality control of mapping quality, and coverage analysis. Non‐synonymous somatic mutations and splice site mutations and copy number variant (CNV) were identified by the Ion Reporter Server System (Thermo Fisher Scientific), and buffy coat DNA was used as a reference to identify variants in DNA of LVLs and background mucosa, as previously reported. Mutations were filtered according to the following criteria to identify high‐confidence somatic mutations: (a) the minimum count for mutant allele reads ≥5 in LVLs or background mucosa samples (b) coverage depth ≥10 at the somatic variant site in samples (c) high‐confidence variant call (Confident Somatic Variants =In), variant allele faction ≥1% and *p*‐value cut‐off 0.05 (d) variants present in the dbSNP (version 138) database are filtered out (UCSC Common SNPs =Not In) (e) no variant allele reads in the buffy coat. This analysis method was reported in the past study, we think this data analyzation is reasonable^20^ Each identified mutant gene was analyzed whether it was oncogenic or not concerning OncoKB (http://oncokb.org/). Oncogenic and likely oncogenic mutations were defined as putative driver mutations. CNVs were examined with confidence level 20 or more and precision level 10 or more. Furthermore, we performed Duplex quantitative real‐time PCR (real‐time qPCR) using the FAM/MGB‐labeled TaqMan probes for TP53 (Hs06423639_cn) and NOTCH1(Hs03718159_cn), and VIC/TAMRA‐labeled TaqMan CNV RNAse P (#4403326) was used for the internal control. NTC reactions were also used to identify PCR contamination. All real‐time qPCR reactions were performed in quadruplicate with gDNA according to the manufacturer's protocol using the StepOne Real‐Time PCR system (Life Technologies, Foster City, CA, USA). The copy number of each sample was estimated by CNV analysis using Copy Caller Software V2.1 (Life Technologies, Foster City, CA, USA).

PyClone analysis, a statistical model for inference of clonal population structures in cancers, was simultaneously performed in all cases. It is a Bayesian clustering method for grouping sets of deeply sequenced somatic mutations into putative clonal clusters while estimating their cellular prevalences and accounting for allelic imbalances introduced by segmental copy number variants and normal‐cell contamination. The source code for PyClone 0.13.0 hosted at https://github.com/aroth85/pyclone was utilized in this study.^22^

### 2.5 Statistical analysis

The number of somatic mutations between LVLs and background mucosa was compared using Fisher's exact test. The comparison of the allele frequency (AF) between the mutation of LVLs and background mucosa was performed with Mann–Whitney U test and Kruskal–Wallis test. Univariate analysis for AF between each risk factor was performed by using polytomous logistic regression analysis. All statistical analyses were performed using SPSS 23.0 (SPSS Inc., Chicago, IL, USA), and a *p*‐value of <0.05 was considered to be statistically significant in two‐sided tests.

## 3 RESULTS

### 3.1 Patient characteristics

Summarized information about patient characteristics and pathologic findings can be seen in Table 1. The mean age of patients at initial diagnosis was 70.1 ± 9.4 years. More men were diagnosed than women (18:7). Of these patients, nine had ESCC, six had IN, and 11 had neoplasia. Tissue sampling methods were a biopsy, ESD, and endoscopic mucosal resection using a cap‐fitted endoscope (EMR‐C) (19 cases, 4 cases, and 2 cases, respectively). Nineteen patients had a smoking habit, and 22 had a drinking habit. Twenty‐three patients had multiple LVLs.

**TABLE 1:** Patient characteristics and pathological findings

Age, years (Mean ±SD)	70.1 ± 9.4
Gender (Male/Female)	18/7
Pathological findings (SCC/IN/Hyperplasia)	9/6/10
Sample (Biopsy/ESD/EMR‐C)	19/4/2
Smoking habit (Yes/No)	19/6
Drinking habit (Yes/No)	22/3
HNSCC (Yes/No)	12/13
Multiple LVLs (Yes/No)	23/2

Abbreviations: EMR‐C, endoscopic mucosal resection using a cap‐fitted endoscope; ESD, endoscopic submucosal dissection; HNSCC, head and neck squamous cell carcinoma; IN, intraepithelial neoplasia; LVLs, Lugol‐voiding lesions; SCC, squamous cell carcinoma; SD, standard deviation.

### 3.2 The amount of DNA extracted from each tissue

The amount of DNA extracted from LVLs and background in each pathological group is shown in Figure S2. There was no significant difference in DNA content between each group.

### 3.3 NGS analysis data

#### 3.3.1 Total detected mutations

**FIGURE 1:**
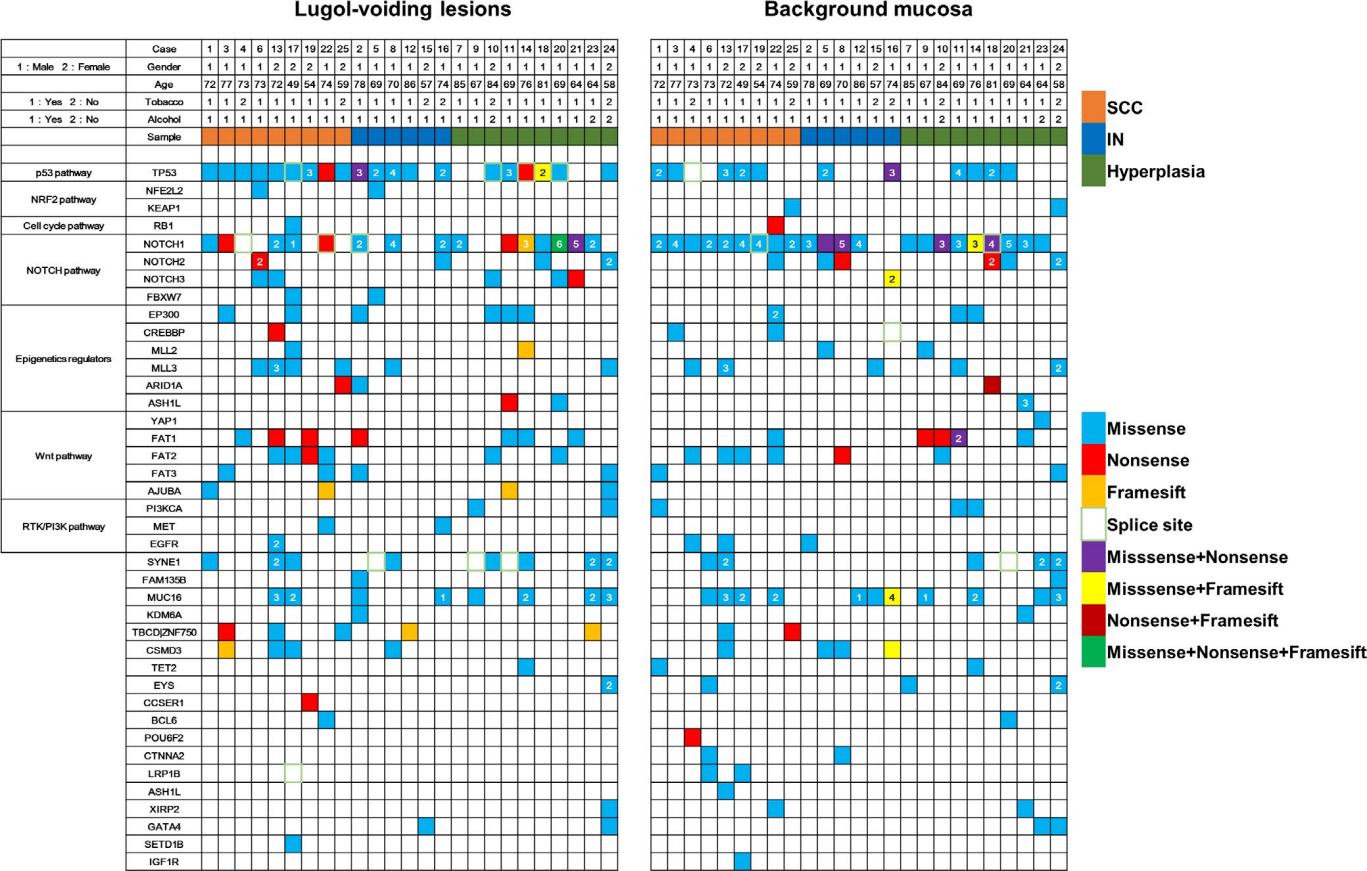
The mutational landscape of somatic alterations. Significantly mutated genes are ranked in the left of the panel and grouped by each pathway. Gender, age, smoking habit, drinking habit, and sample are shown at the top of the panel. This figure shows *TP53* and *NOTCH1* mutations are common in LVLs and background mucosa, and background mucosa has numerous somatic mutations similar to LVLs

Targeted NGS was performed for 25 cases, 75 specimens (LVLs, background mucosa, and buffy coat from each patient). We confirmed at least one somatic mutation in all specimens of LVLs and background mucosa. One hundred eighty‐eight mutations of 36 genes in LVLs were identified and 199 of 34 genes in background mucosa (Figure 1, Table 2). In three cases (33%) in SCC, one case (17%) in IN, and three cases (30%) in hyperplasia, overlapped somatic mutations were observed in LVLs and background mucosa.

**TABLE 2:** Analysis data by the next‐generation sequencer

	LVLs	Background mucosa	*p* value
Mutant genes, *n*	36	34	
Somatic mutations, *n*	188	199	
Coverage, median (range)	242 (35–1757)	304 (27–1705)	<0.001*
AF, % (range)	13 (3–99)	7 (3–100)	0.003*
Missense, *n* (%)	144 (76)	171 (86)	0.0192*
Nonsense, *n* (%)	22 (12)	18 (9)	0.4085
Frameshift, *n* (%)	10 (5)	4 (2)	0.1038
Non‐frameshift deletion, *n* (%)	1 (0.5)	0 (0)	0.4858
Splice site, *n* (%)	11 (6)	6 (3)	0.2173
Putative driver mutations, *n* (%)	40 (21)	29 (15)	
Coverage, median (range)	225 (43–649)	253 (27–681)	0.215
AF, % (range)	17 (4–88)	6 (3–30)	0.0002*

Abbreviations: AF, allele frequency; LVLs, Lugol‐voiding lesions

* p<0.05

#### 3.3.2 The mutational analysis between LVLs and background mucosa

**FIGURE 2:**
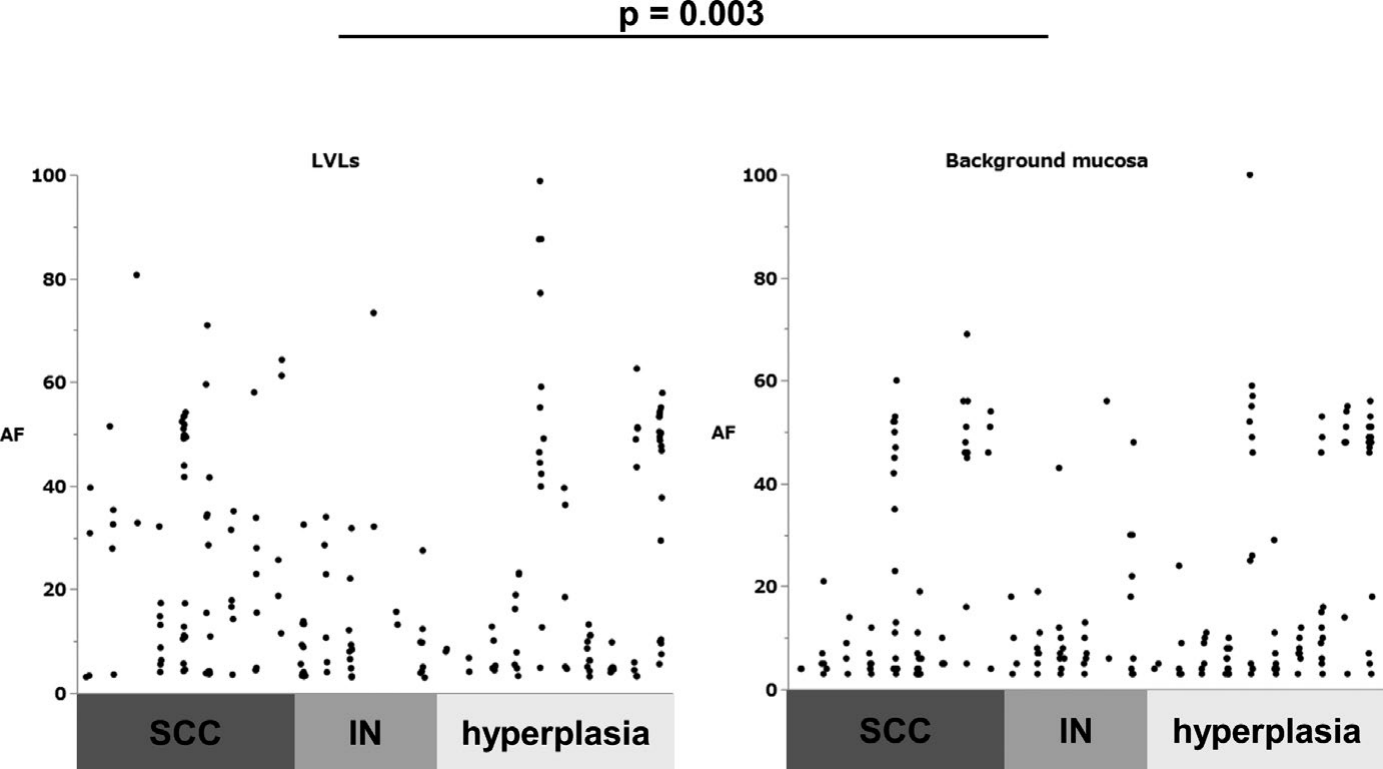
(A) AF of all mutations in LVLs and background mucosa. AF in LVLs (SCC, IN, and hyperplasia) is significantly higher than background mucosa. (B) AF of putative driver mutations in LVLs and background mucosa. AF in LVLs (SCC, IN, and hyperplasia) is significantly higher than in background mucosa

There was not any significant difference between the rate of the case which had at least one putative driver in LVLs and background mucosa (15% vs. 20%, Fisher exact test, *p* = 0.08), on the other hand, AF of all mutations in LVLs was significantly higher than in background mucosa (13 [range, 3–99] vs. 7 [range, 3–100], Mann–Whitney U test, *p* = 0.003) (Figure 2). Moreover, AF of the putative driver in LVLs was significantly higher than in background mucosa (17 [range, 4–88] vs. 6 [range, 3–30], Mann–Whitney U test, *p* = 0.0002).

#### 3.3.3 Intra‐group analysis in all mutation

**FIGURE 3:**
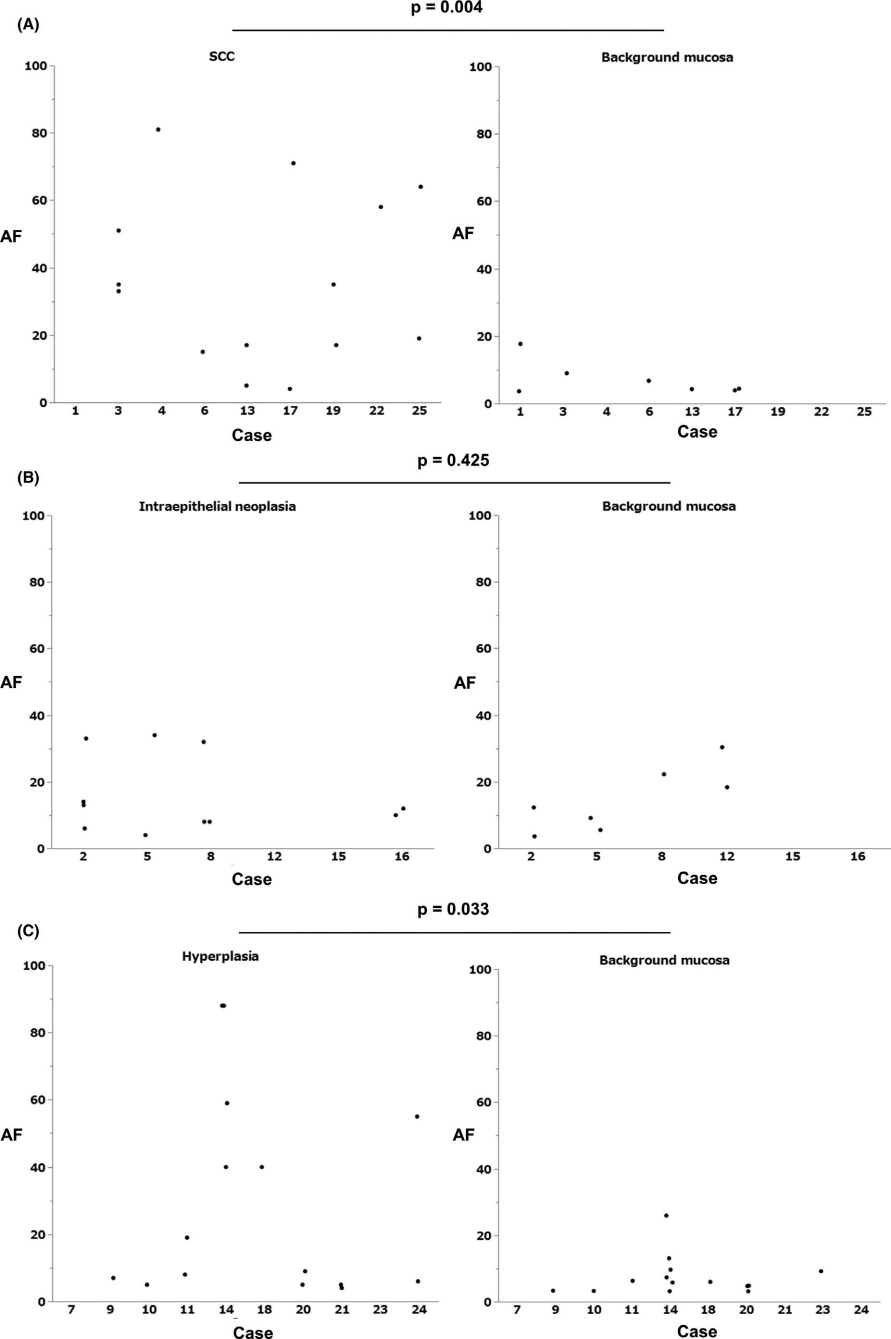
(A) AF of putative driver mutations in SCC and background mucosa with SCC. AF in SCC is significantly higher than in their background mucosa. (B) AF of putative driver mutations in IN and background mucosa with IN. There is no significant difference between the AF in IN and background mucosa. (C) AF of putative driver mutations in hyperplasia and background mucosa with hyperplasia. AF in hyperplasia is significantly higher than background mucosa

Intra‐group analysis of SCC, IN, and hyperplasia in all mutations showed that SCC had accumulated mutations, which is significant (19 [range, 3–81] vs. 8 [range, 3–60], Mann–Whitney U test, *p* = 0.016) (Figure S3A). There was no such tendency in the group of IN (9 [range, 3–73] vs. 6 [range, 3–30], Mann–Whitney U test, *p* = 0.146) (Figure 3B), and hyperplasia (12 [range, 3–99] vs. 9 [range, 3–100], Mann–Whitney U test, *p* = 0.126) (Figure S3C).

#### 3.3.4 Intra‐group analysis in putative driver

Intra‐group analysis of SCC, IN, and hyperplasia in putative driver showed that SCC had accumulated mutations in the putative driver, which is significant (34 [range, 4–81] vs. 4 [range, 4–18], Mann–Whitney U test, *p* = 0.004) (Figure 3A). There was no such tendency in the group of IN (12.5 [range, 4–34] vs. 11 [range, 4–22], Mann–Whitney U test, *p* = 0.425) (Figure 3B). The group of hyperplasia had a similar tendency in the putative driver (9 [range, 4–88] vs. 6 [range, 3–26], Mann–Whitney U test, *p* = 0.033) (Figure 3C).

#### 3.3.5 Analysis of *TP53* mutations and *NOTCH1* mutations

In LVLs, *TP53* was the most dominant mutation and identified in 20 (SCC 9/IN 5/hyperplasia 6) cases (80%). Among them, 17 (7/4/6) cases (85%) had the putative driver mutation. On the other hand, in background mucosa, the *TP53* mutation was identified in 12 (6/2/4) cases (48%). Of them, 10 (4/2/4) cases (91%) had the putative driver mutation. The rate of the case which had *TP53* mutations was significantly higher in LVLs than in background mucosa (80% vs. 48%, *p* = 0.037, Fisher exact test).

In background mucosa, *NOTCH1* was the most dominant mutation and identified in 22 (SCC 9/IN 4/hyperplasia 9) cases (88%). Among them, four (0/1/3) cases (18%) had the putative driver mutation. On the other hand, in LVLs, the *NOTCH1* mutation was identified in 15 (5/3/7) cases (60%). Of these, five (1/0/4) cases (66%) had the putative driver mutation. The rate of the case which had *NOTCH1* mutations was significantly higher in background mucosa than in LVLs (88% vs 64%, *p*=0.029, Fisher exact test). We have illustrated the details of somatic mutations of *TP53* and *NOTCH1* in Supplementary Figure 4. Seventy‐two percent (23/32) of the *TP53* mutations in LVLs and 73% (16/22) in background mucosa were due to the putative driver, and 20% (7/35) of *NOTCH1* mutations in LVLs and 8% (5/61) of background mucosa were due to the putative driver.

**FIGURE 4:**
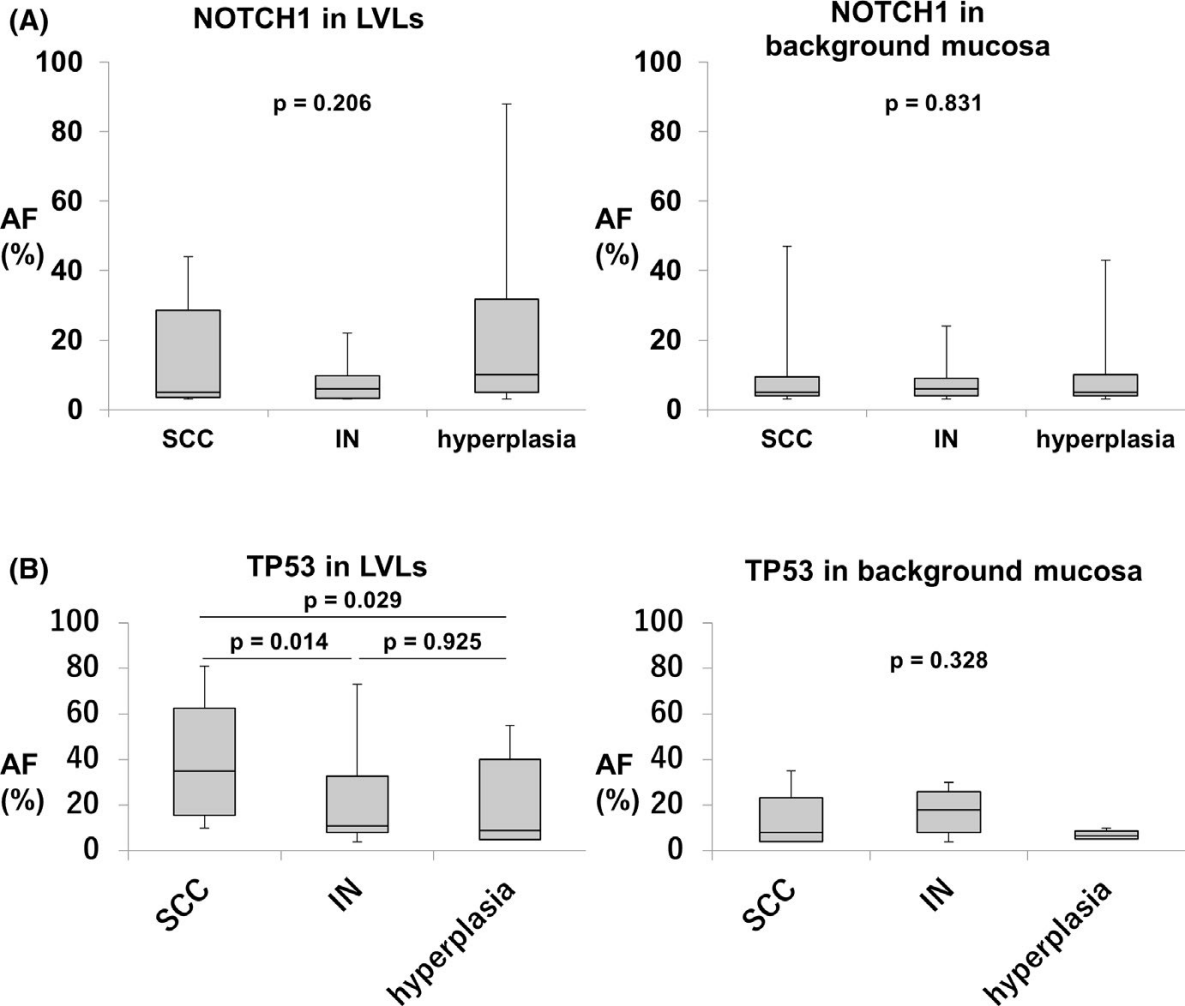
(A) AF of the *NOTCH1* mutation in LVLs and background mucosa. There was not a significant difference in AF between LVLs in patients with SCC, IN, and hyperplasia. This result was similar to background mucosa. (B) AF of the *TP53* mutation in LVLs and background mucosa. There was significantly higher AF in SCC than in IN and hyperplasia, but not in background mucosa

Focusing on *NOTCH1*, there was not a significant difference in AF between LVLs in patients with SCC, IN, and hyperplasia (5 [range, 3–44], 6 [range, 3–22], and 10 [range, 3–88], respectively). This result was similar to background mucosa (5 [range, 3–47], 6 [range, 3–24], and 6 [range, 3–43], respectively) (Figure 4A). On the other hand, focusing on *TP53*, there was significantly higher AF in SCC than in IN and hyperplasia (35 [range, 10–85] vs. 11 [range, 4–73], *p* = 0.014, 35 [range, 10–85] vs. 9 [range, 5–55], *p* = 0.029, Kruskal–Wallis test), but not in background mucosa between groups (8 [range, 4–35], 18 [range, 8–30], and 6.5 [range, 5–10], respectively) (Figure 4B).

Finally, we show the other mutational status of LVLs and background mucosa in Figure S5.

#### 3.3.6 Intra‐group analysis in *TP53* and *NOTCH1* mutations

**FIGURE 5:**
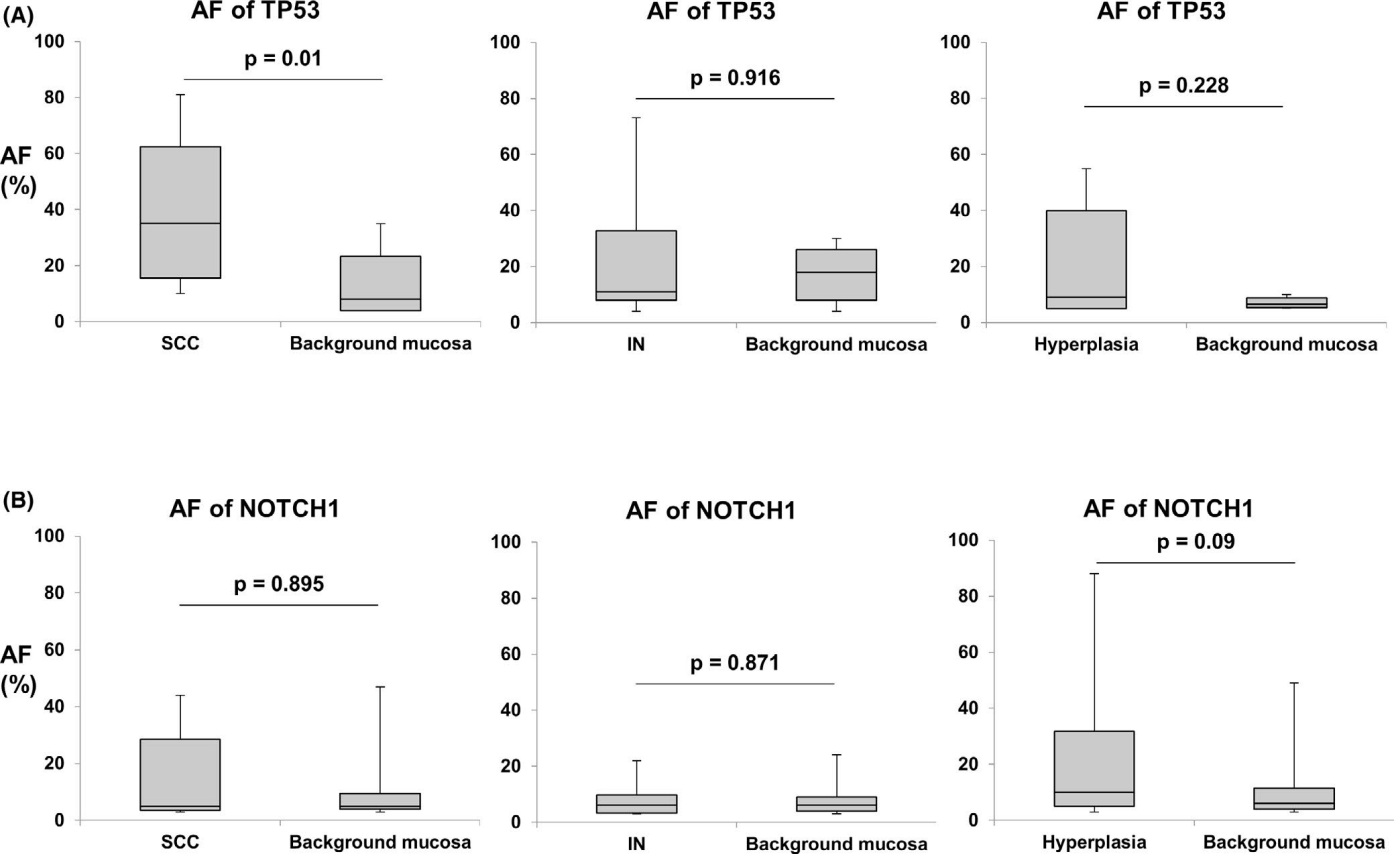
(A) AF of the *TP53* mutation intra‐group of SCC, IN, and hyperplasia. Only SCC had significantly higher AF of *TP53* mutations than in its background mucosa. (B) AF of the *NOTCH1* mutation intra‐group of SCC, IN, and hyperplasia. No significant difference was not observed between LVLs and its background mucosa

Intra‐group analysis of SCC showed that AF of *TP53* mutations was significantly higher in LVLs than in background mucosa (35 [range, 10–81] vs 8 [range, 4–8], *p* = 0.009, Mann–Whitney U test), but not in IN and hyperplasia. As for *NOTCH1*, a significant difference was not observed between LVLs and background mucosa in each group (Figure 5).

#### 3.3.7 AF of putative driver in patients with or without HNSCC and SCC

There was a significant difference between AF of putative driver mutation from the background mucosa in patients with HNSCC (9 [range, 3–30] vs. 4 [range, 3–9], *p* = 0.017, Mann–Whitney U test). However, a significant difference was not identified between patients with SCC, IN, and hyperplasia (8 [range, 4–30] vs. 6 [range, 3–26], *p* = 0.248, Mann–Whitney U test) (Figure S6).

#### 3.3.8 AF of background mucosa under the classification of drinking/smoking grade

The drinking grade was divided into less than 40 g/day or more,^23^ and the smoking grade was divided into less than a 30 pack‐year or more.^24^ The relationship between drinking/smoking grade and mutation status in background mucosa is in Figure S7. There was a significant difference between heavy drinker/ heavy smoker and heavy drinker/ light smoker or light drinker/ heavy smoker.

Univariate analysis of the effects of drinking, smoking, and age on AF of the background mucosa was also performed. There was no significant difference (Table S2).

#### 3.3.9 Other putative driver mutations

Other putative driver mutations such as *EP300*, *CREBBP*, *ARID1A* in epigenetics regulators, *FAT1* in the Wnt pathway, and *PI3KCA* in the *RTK*/*PI3 K* pathway are also found from LVLs or background mucosa.

#### 3.3.10 Copy number variants

In LVLs, there were 34 CNVs in 15 cases, and in background mucosa there were three CNVs in one case (Table S3). At the somatic mutation point, two cases in LVLs had one CNV for each, and no case in background mucosa had. The two CNVs were TP53 in case 4 and MET in Case 22 whose AF was 81% and 16%. PyClone analysis was performed in all cases and confirmed that the prevalence of cell frequency was almost equivalent to the value of AF (Figure S8).

Especially, CNV of TP53 and NOTCH1 was validated by Duplex quantitative real‐time PCR, suggesting that CNV in these regions was almost not identified (Table S4).

#### 3.3.11 The mutational analysis in healthy controls

Of five healthy controls, two controls have no somatic mutation and three controls have some somatic mutations without the putative driver. We showed the detail about the mutation in healthy controls in Table S5 and Figure S9.

## 4 DISCUSSION

To the best of our knowledge, this is the first study to prove the gene mutation in the early stages of the ESCC carcinogenic process with mutational analysis of background mucosa. We proved that the background mucosa in patients with a high risk of ESCC has numerous gene mutations similar to those of SMGs in advanced cancer. Surprisingly, some putative driver mutations as *TP53*, *NOTCH1*, *ARID1A*, *PIK3CA*, *EP300*, and *FAT1* were already recognized in the background mucosa. In contrast, two healthy controls two healthy controls have no somatic mutations in their esophageal mucosa. *NOTCH1* mutation was detected in three healthy controls. All of them were not the putative driver mutations. Unlike the population with a high risk of ESCC, there were some cases with no somatic mutation in the background mucosa. Judging from these results, it was assumed that exposure to risk factors would increase the somatic mutation in the background mucosa. These results indicate that putative drivers cause carcinogenesis. The fact that there were few common mutations between LVLs and background mucosa within the same case also matches this concept because field cancerization causes the development of independent cancer synchronously or metachronously from background mucosa.

The NOTCH pathway is involved in cell growth and apoptosis.^25^ The mutation of the NOTCH pathway has been implicated in various cancers, including ESCC.^26^, ^27^ In ESCC, the frequency of *NOTCH1* mutation was reported to be higher in the early stages of carcinogenesis than in the advanced stages, suggesting *NOTCH1* could be an earlier mutation of ESCC.^28^ In this study, the somatic mutation of *NOTCH1* in the background mucosa was dominantly observed; there is a possibility that the *NOTCH1* mutation was contained before the neoplastic change. The rate of *NOTCH1* somatic mutation was significantly lower in LVLs (also in SCC) than in background mucosa. Furthermore, inter‐group analysis of SCC, IN, and hyperplasia, between LVL and background mucosa the *NOTCH1* mutation did not show any difference in AF. Yokoyama A et al. reported that *NOTCH1* mutation significantly increases in number with heavy smoking and drinking in esophageal SCC patients in Japan.^29^ It seems that the *NOTCH1* mutation plays an important role in the development of SCC. But, *NOTCH1* mutation was reported to be more frequent in background mucosa than SCC. This phenomenon was similar in our results, moreover, we confined there was no frequency or AF amplification. Judging from these results, though somatic mutation of *NOTCH1* emerges in iodine‐stained mucosa, somatic mutation of *NOTCH1* alone would not lead to SCC.

Putative driver mutations of *TP53* were identified mostly in both LVLs and background mucosa. *TP53* mutation is known as a frequent tumor suppressor gene and is present in more than 50% of human cancers.^30^, ^31^ *TP53* mutations have been shown to be an early event of ESCC and are associated with cancer progression and a poor prognosis.^32^, ^33^, ^34^ AF of the *TP53* mutation increased significantly from hyperplasia to SCC. In addition, the rate of *TP53* somatic mutation was higher in SCC than in background mucosa. Besides, the intra‐group analysis revealed that SCC has significantly higher AF than its background mucosa. These facts indicate that somatic mutation of *TP53* is the main driver of SCC.

The difficulty was often occurred to clearly distinguish between carcinoma in situ, IN, and hyperplasia. In this study, the frequency of TP53 mutation was 100%/83%/60% in SCC/IN/Hyperplasia, respectively, suggesting that TP53 mutation does not always indicate SCC, but the absence of TP53 mutation may indicate that it is not SCC. On the other hand, the frequency of the absence of NOTCH1 mutation with TP53 mutation was 33%/40%/11% in SCC/IN/Hyperplasia. The usefulness of the absence of NOTCH1 mutation with TP53 mutation for diagnosis of ESCC was not obvious, but it could be used to distinguish between neoplastic and non‐neoplastic changes.

Past studies reported that various CNVs have been reported not only in SCC but also IN, hyperplasia, and background mucosa.^6^, ^14^, ^16^, ^29^, ^35^ We also detected some CNVs in those categories. However, the number of CNV identified by NGS was relatively few compared to the previous reports. We estimate that the result was caused by targeting early stage cancer and analyzing limited regions under the target sequence. Therefore, the additional method by real‐time PCR was used to validate CNVs in TP53 and NOTCH1. Amplification in TP53 was observed in only two cases of LVL and two cases of BM, and deletion in TP53 was observed in only two cases of LVL. Amplification in NOTCH1 was observed in only one case of LVL, and deletion was observed in only one case of LVL. These results were not significantly different from the previous reports.

Around 45% of lesions in IN is missed under white light without Lugol staining.^36^ Lugol chromoendoscopy is reported to be very useful in detecting SCC and IN.^37^, ^38^ Also, by Lugol staining, the carcinogenic risk can be evaluated by the severity of LVLs, but Lugol staining is a painful and complicated examination, and it would be impossible to perform it as routine in all cases. On the other hand, there were also putative driver mutations in iodine‐stained mucosa. In this study, the AF in putative driver mutations in background mucosa was significantly higher in patients with HNSCC than without HNSCC. Previous studies that reported the high incidence of ESCC in patients with HNSCC were confirmed.^39^ In other words, among specific high‐risk patients of ESCC, AF of putative drivers was significantly higher. It would be possible to evaluate the risk of carcinogenesis more accurately by analyzing mutational characteristics of background mucosa over time. Furthermore, in this study, all the tissue of all cases included in this study was obtained by biopsy or therapeutic endoscopic resection. Therefore, it is easy to collect the tissue and would be practical.

Gene mutations in ESCC have been reported by past several studies.^14^, ^15^, ^16^, ^21^, ^40^, ^41^, ^42^ However, most of the previous studies targeted advanced cancer, but our study focused on background mucosa as well as on superficial SCC, IN, and hyperplasia. We could capture very early gene mutations of carcinogenesis. Identified gene mutations were similar to those present in advanced cancer. Moreover, these mutations were confirmed not only in superficial cancer but also IN, and hyperplasia.

This study has some limitations. First, we had a small sample size. Second, we focused only on high‐risk patients. Therefore, there was no information on healthy controls. Third, this study was evaluated at one point in time; for more accurate evaluation, observation over a long period is needed. Forth, since microdissection was not performed on biopsy samples, it cannot be ruled out that IN or hyperplastic samples may contain various types of cells. Fifth, the method of evaluation of CNA in this study was different from that of the previous report.

In conclusion, the background mucosa in patients with LVLs already has accumulated gene mutations. Notably, many putative driver mutations were confirmed in *TP53* and *NOTCH1*. The *TP53* could be the main target gene of the carcinogenesis in esophageal SCC; this mutation was also found in the background mucosa in high‐risk patients. *NOTCH1* could be one of the early mutations of carcinogenesis but estimated not to be the target gene.

## CONFLICT OF INTEREST

The authors have no conflict of interest.

## ETHICS APPROVAL

This study was approved by the Bioethics Committee of Chiba University Hospital (No. 826). We obtained written informed consent from all patients in this study.

## Supporting information

Fig S1‐S9

Table S1‐S5

## Data Availability

The author elects to not share data.
