# Supplementary material for: Comprehensive mutational analysis of background mucosa in patients with Lugol‐voiding lesions
Source: Cancer Med. 2021 May 2;10(11):3545–55. doi: 10.1002/cam4.3905 (PMC8178505; doi:10.1002/cam4.3905)
Supplement: Supplementary file 1 — Fig S1‐S9 [file CAM4-10-3545-s002.docx]

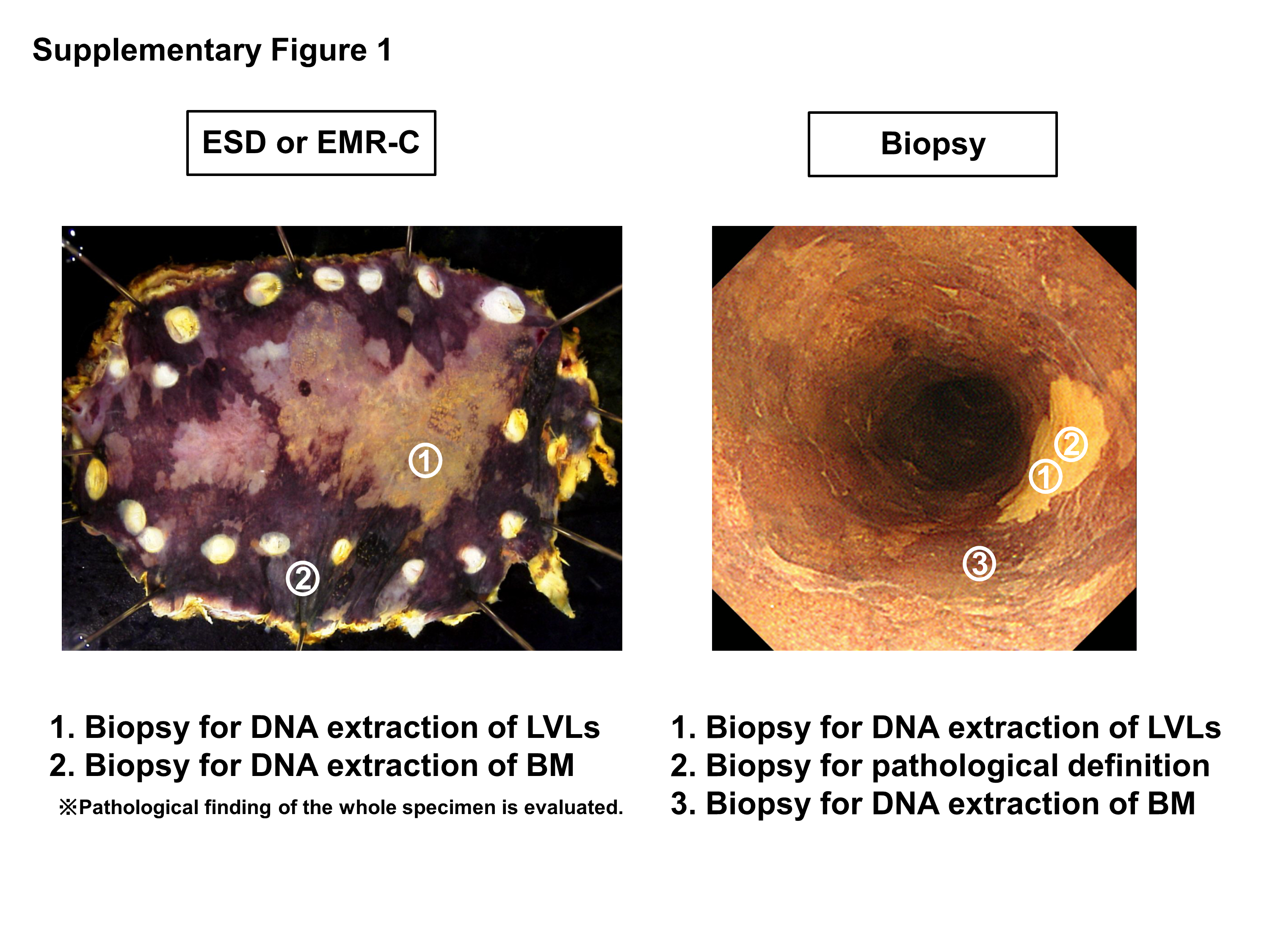


Supplementary Figure 1: Biopsy locations of LVLs and background mucosa

Each tissue was obtained from this location after lugol spray to clarify the border line between LVLs and background mucosa.


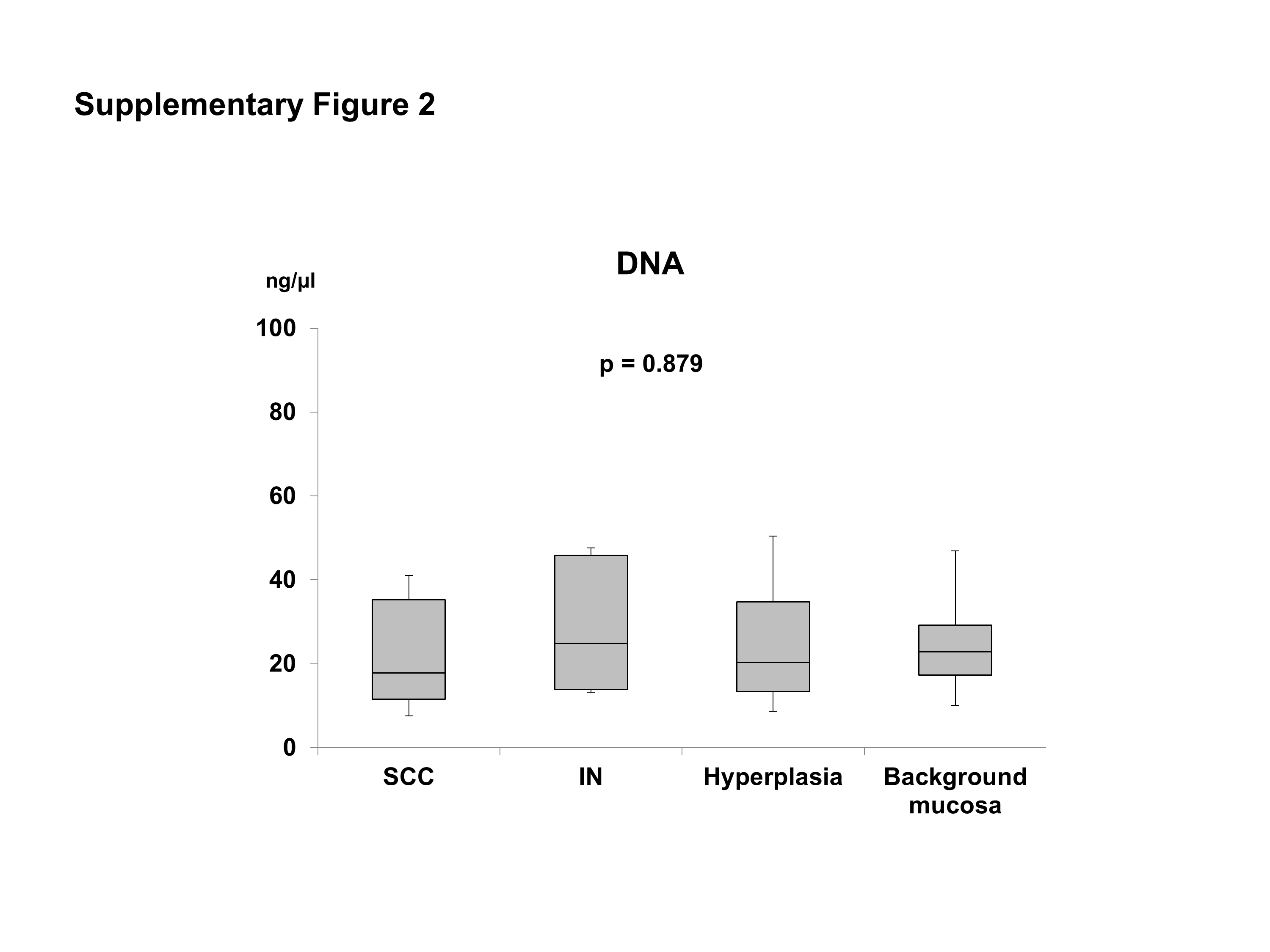


Supplementary Figure 2: The amount of DNA extracted from LVLs and background mucosa

There was no significant difference in DNA content between each group.


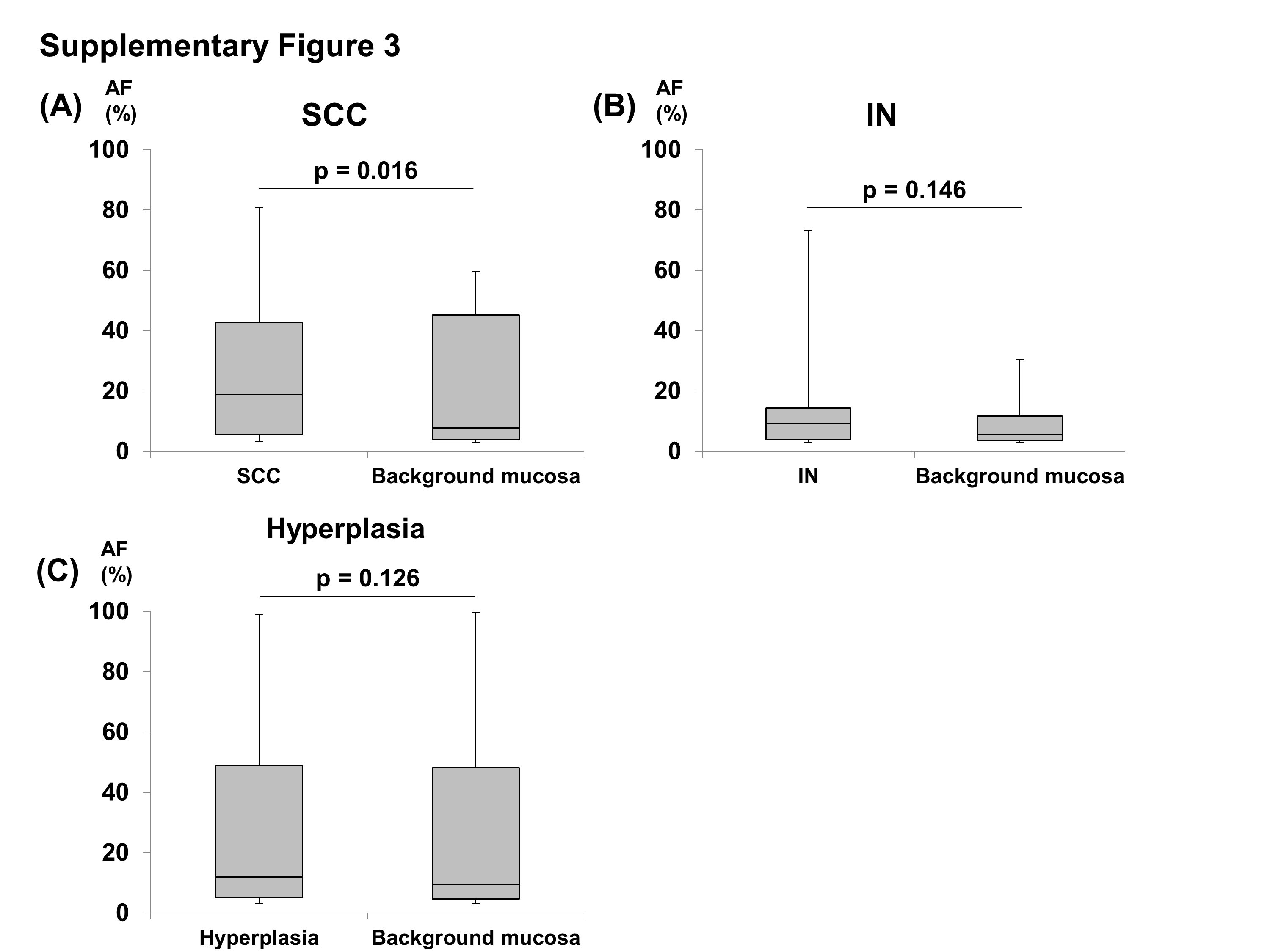


Supplementary Figure 3: (A) AF of all mutations in SCC and background mucosa with SCC

AF in SCC is significantly higher than in their background mucosa.

(B) AF of all mutations in IN and background mucosa with IN

There is no significant difference between the AF in IN and background mucosa.

(C) AF of all mutations in hyperplasia and background mucosa with hyperplasia

AF in hyperplasia is significantly higher than background mucosa.


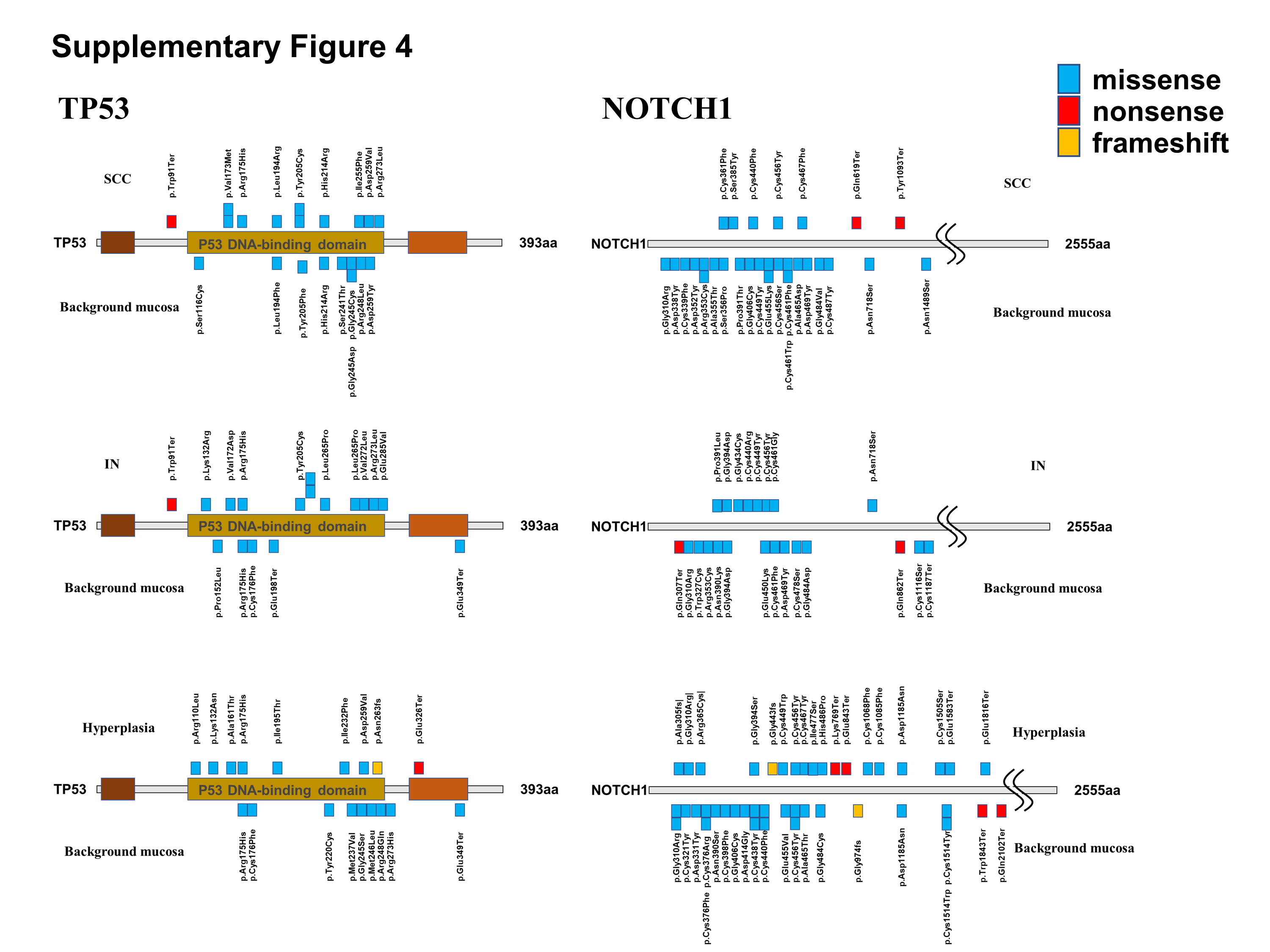


Supplementary Figure 4: Distribution of somatic mutations in TP53 and NOTCH1 in LVLs (above), and background mucosa (below).


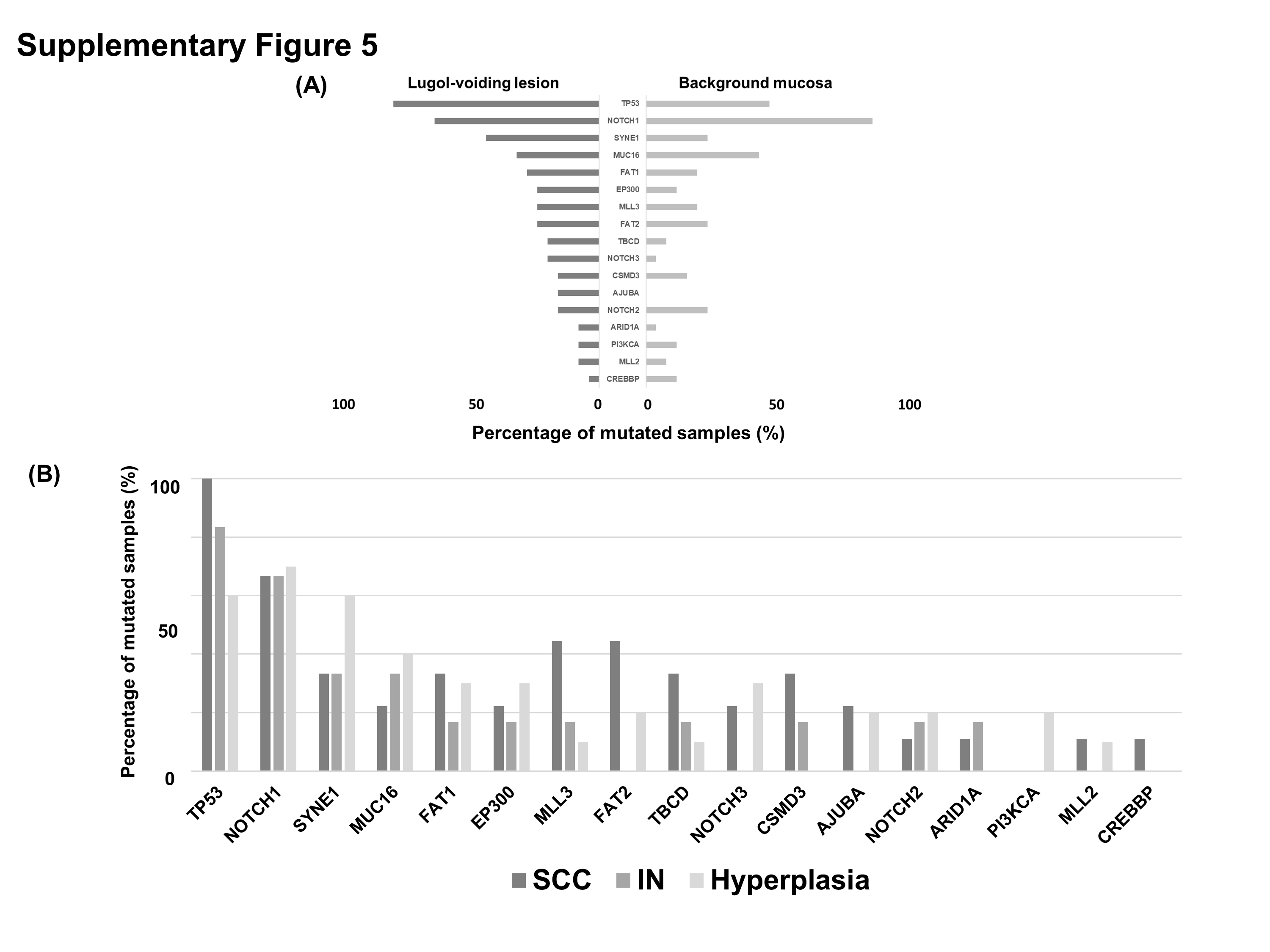


Supplementary Figure 5: (A) Mutational status of LVLs and background mucosa

NOTCH1, MUC16 and NOTCH2 mutations are more frequently in background mucosa than in LVLs.

(B) Mutated genes identified in each LVL

TP53 mutation was detected in all SCC cases. Each LVL has similar genetic mutations.


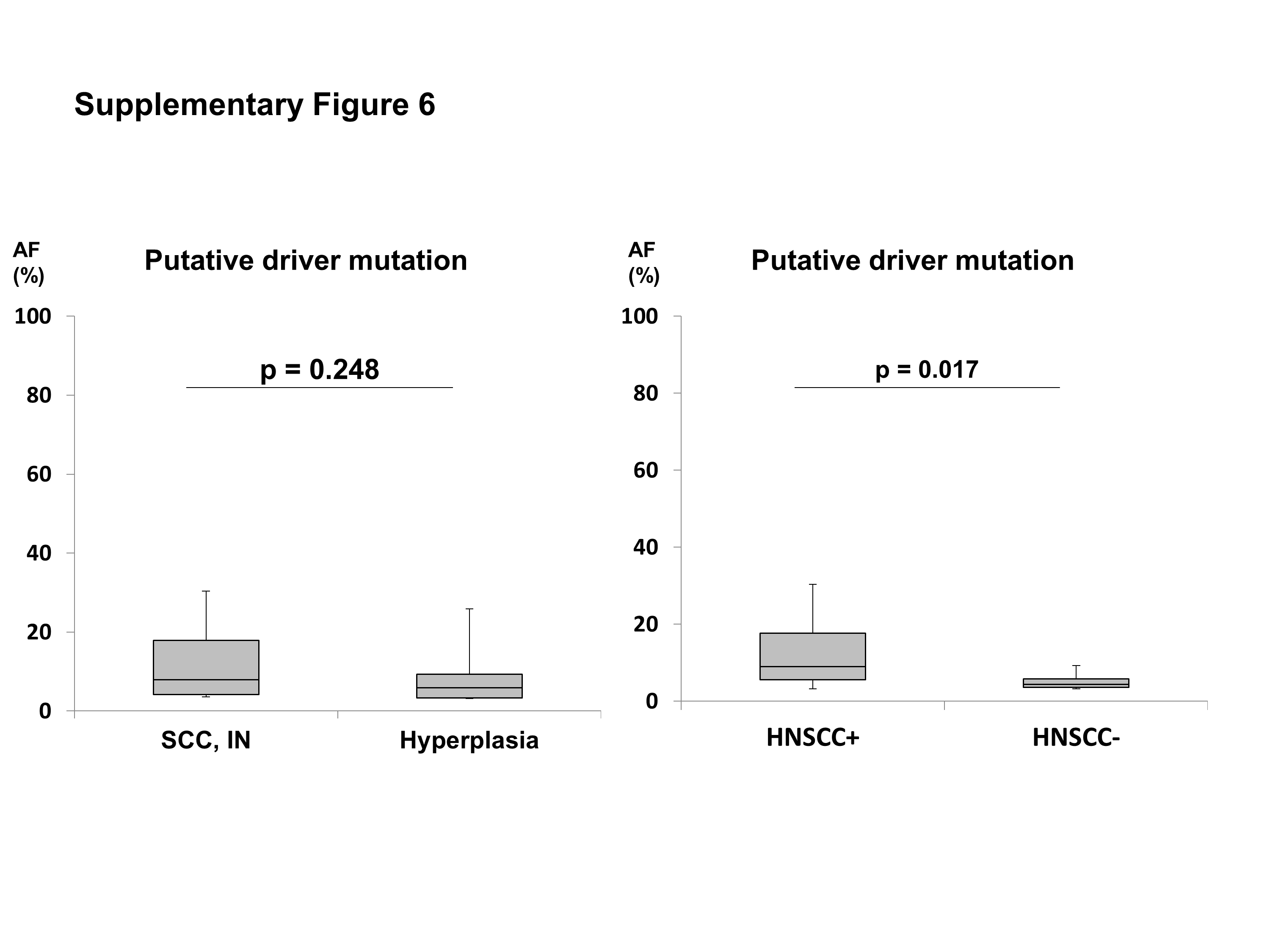


Supplementary Figure 6: Putative Driver Mutation in Background Mucosa

There was significantly higher AF of putative driver mutations in the background mucosa in patients with HNSCC than without HNSCC. However, it was not identified between patients with SCC/IN and hyperplasia.


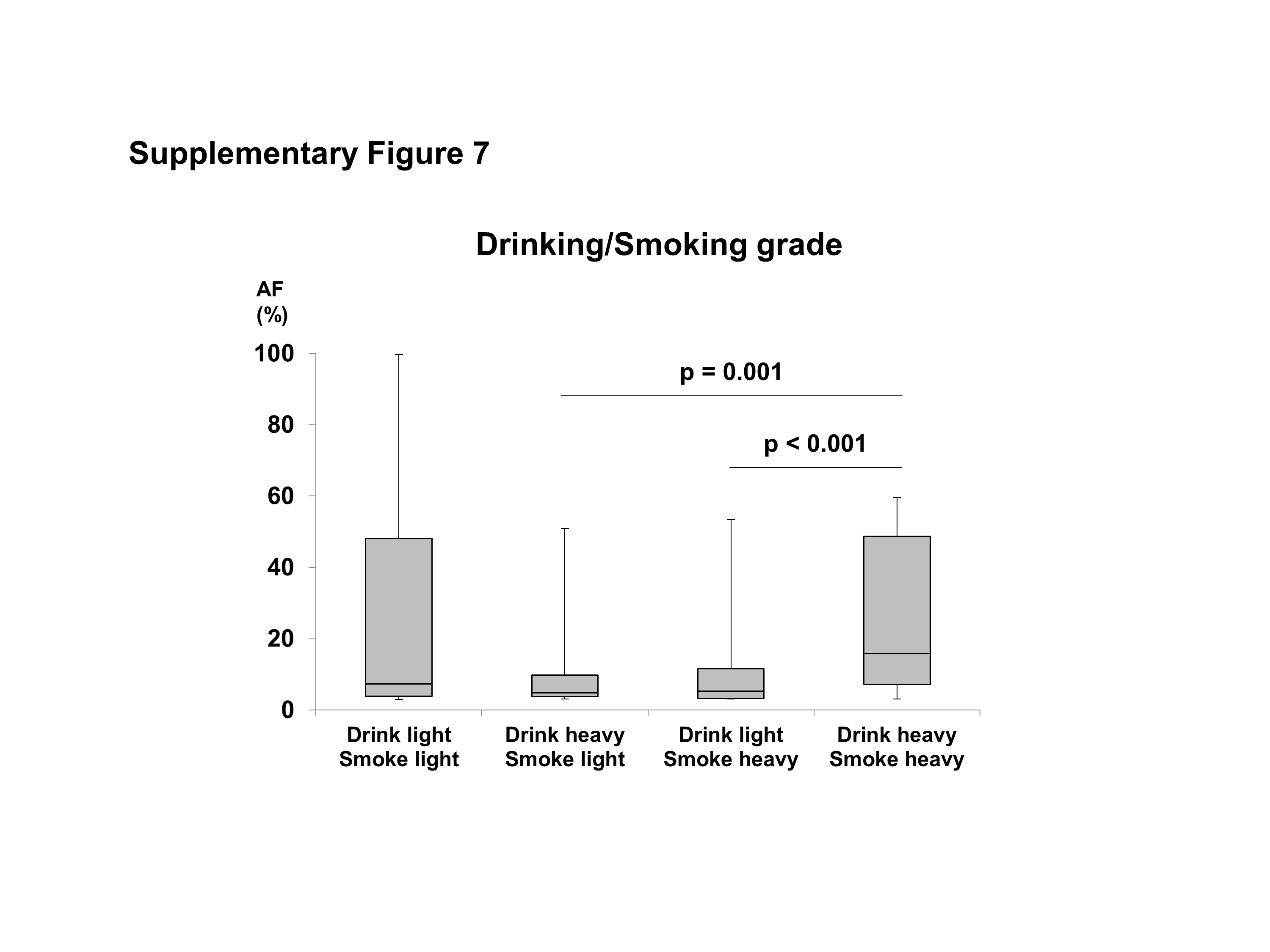


Supplementary Figure 7: AF of background mucosa under the classification of drinking/smoking grade

There was significantly higher AF in heavy drinker/ heavy smoker than heavy drinker/ light smoker or light drinker/ heavy smoker.


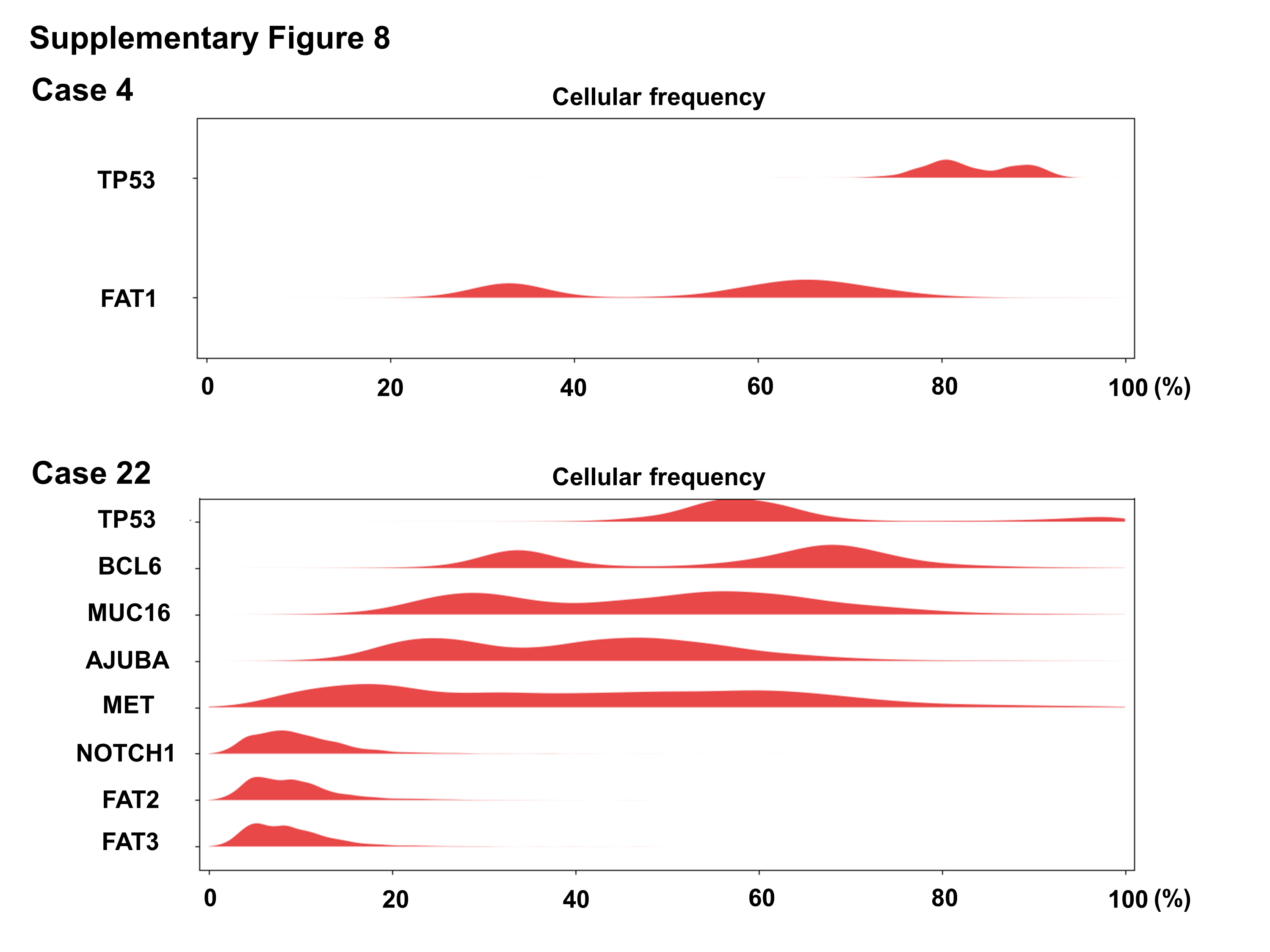


Supplementary Figure 8: The prevalence of cellular frequency

PyClone analysis was performed and the prevalence of cellular frequency was almost equivalent to the value of AF.


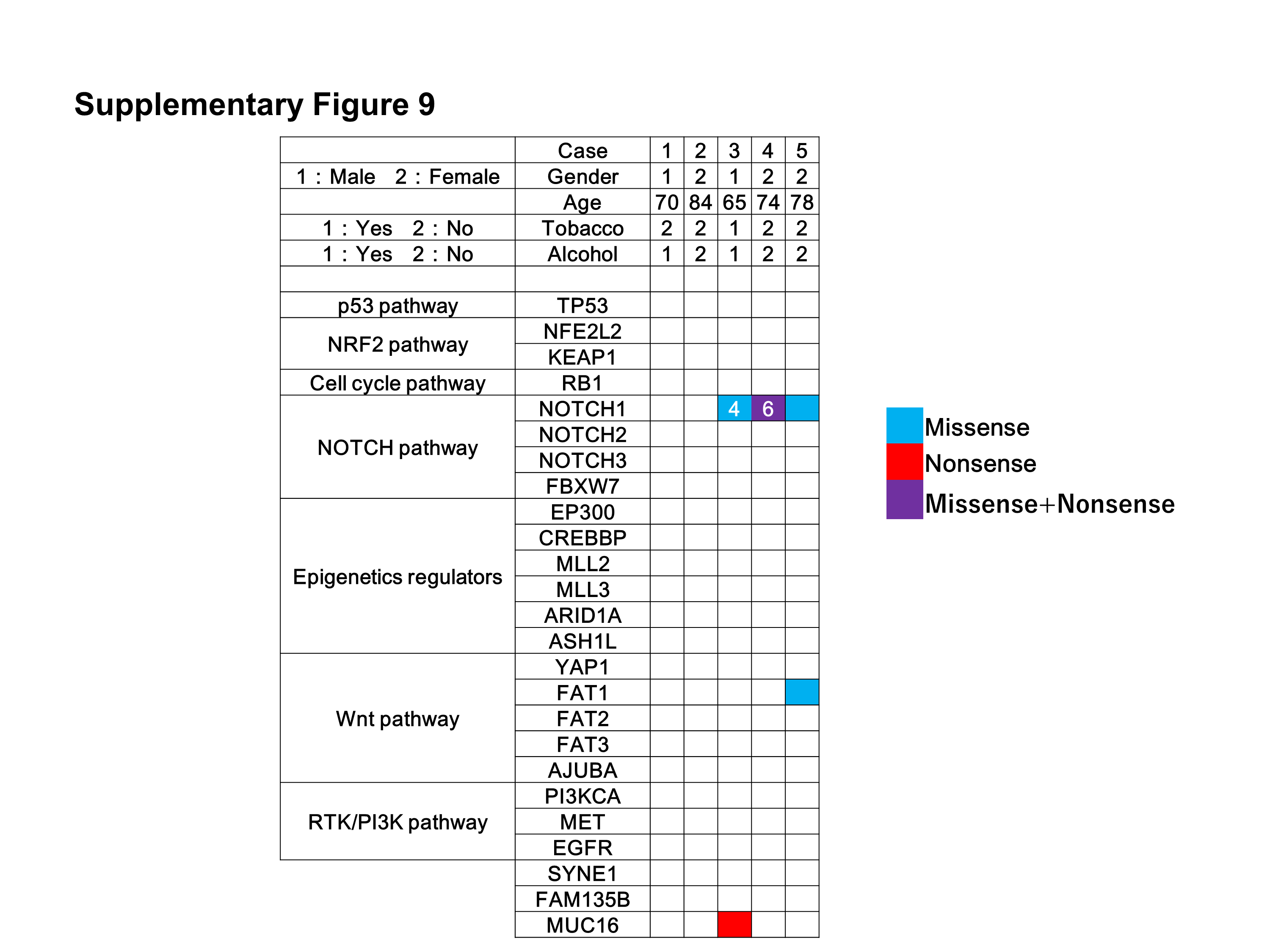


Supplementary Figure 9: The mutational landscape of somatic alterations in healthy controls

This figure shows NOTCH1 mutations are common in healthy controls. They have fewer somatic mutations compare to those who have risk of SCC.
