# Supplementary material for: Comprehensive mutational analysis of background mucosa in patients with Lugol‐voiding lesions
Source: Cancer Med. 2021 May 2;10(11):3545–55. doi: 10.1002/cam4.3905 (PMC8178505; doi:10.1002/cam4.3905)
Supplement: Supplementary file 2 — Table S1‐S5 [file CAM4-10-3545-s001.docx]

**Supplementary Table**

**Supplementary Table 1. Seventy significantly mutated genes were included in determining EC panel**

| **TP53** | **NOTCH1** | ***ADAM29*** | ***AJUBA*** | ***ASH1L*** | ***CDKN2A*** | ***CREBBP*** |
| --- | --- | --- | --- | --- | --- | --- |
| ***EGFR*** | ***EP300*** | ***FAM135B*** | ***FAT1*** | ***FAT2*** | ***FAT3*** | ***FBXW7*** |
| ***KDM6A*** | ***KEAP1*** | ***KMT2C*** | ***KMT2D*** | ***NOTCH2*** | ***NOTCH3*** | ***NSD*** |
| ***PIK3CA*** | ***PTEN*** | ***RB1*** | ***SETD1B*** | ***YAP1*** | ***ZNF750*** | ***CSMD3*** |
| ***XIRP2*** | ***STOML3*** | ***CTNNA2*** | ***MUC16*** | ***EYS*** | ***ANO5*** | ***PANK3*** |
| ***SOX2*** | ***BCL6*** | ***MYC*** | ***POU6F2*** | ***KRT8*** | ***PCDH9*** | ***LRP1B*** |
| ***SYNE1*** | ***ATP6V1G3*** | ***KIF1A*** | ***KLF5*** | ***TET2*** | ***FAM190A*** | ***TGFBR2*** |
| ***ARID1A*** | ***SMAD4*** | ***FHIT*** | ***RUNX1*** | ***ERBB2*** | ***MET*** | ***GATA4*** |
| ***GATA6*** | ***VEGFA*** | ***TP63*** | ***CCND1*** | ***SMAD2*** | ***IGF1R*** | ***CDX1*** |
| ***CDX2*** | ***PTGS2*** | ***KRAS*** | ***NFE2L2*** | ***NFE2L2*** | ***CTNNB1*** | ***BRAF*** |

EC, esophageal cancer

**Supplementary Table 2. A univariate analysis of the effects of drinking, smoking, and age on AF of the background mucosa**

|  | **Univariate analysis** | |
| --- | --- | --- |
|  | **p value** | **HR(95%CI)** |
| **Drinking(≧40g/day)** | **0.943** | **0.938(0.161-5.461)** |
| **Smoking(≧30 pack-year)** | **0.157** | **3.929(0.591-26.107)** |
| **Age(≧73 years old)** | **0.943** | **0.938(0.161-5.461)** |

HR, hazard ratio

**Supplementary Table 3A. Copy number alterations in LVLs**

| **Case** | **Sample** | **Iscn** |
| --- | --- | --- |
| **1** | **SCC** | **3q26.32q28(178947181-189612296)x3** |
| **2** | **IN** | **3q26.32q28(178922301-189612296)x3** |
| **4** | **SCC** | **3q26.32(178916549-178952157)x3** |
|  | **SCC** | **3q26.33q28(181430021-189612296)x4** |
|  | **SCC** | **6q25.1q25.2(152443533-152712765)x3** |
|  | **SCC** | **8q23.3q24.23(113236953-139380232)x3** |
|  | **SCC** | **9p21.3(21968185-21994410)x0** |
|  | **SCC** | **13q12.2q22.1(28537226-73650083)x1** |
|  | **SCC** | **17p13.1(7572847-7579960)x1** |
| **5** | **IN** | **3q26.32q28(178916549-189612296)x3** |
| **6** | **SCC** | **3q26.32q28(178916549-189612296)x3** |
| **8** | **IN** | **3q26.32q28(178916549-189612296)x3** |
| **12** | **IN** | **3q26.32q28(178916549-189612296)x3** |
|  | **IN** | **8q23.3q24.23(113256567-139164499)x3** |
|  | **IN** | **9p21.3q34.3(21968185-139438571)x1** |
|  | **IN** | **13q12.2q22.1(28537226-73650083)x1** |
| **14** | **Hyperplasia** | **3q26.32q28(178916549-189612296)x5** |
|  | **Hyperplasia** | **9q34.3(139390438-139438571)x3** |
|  | **Hyperplasia** | **11q14.3q22.2(92085237-102100690)x1** |
|  | **Hyperplasia** | **13q12.2q22.1(28537226-73650083)x1** |
| **17** | **SCC** | **3q26.32q28(178916549-189612296)x4** |
|  | **SCC** | **7q31.2(116339090-116436183)x10** |
|  | **SCC** | **7q36.1(151860233-151879416)x1** |
|  | **SCC** | **9p21.3(21968185-21994410)x1** |
|  | **SCC** | **11q14.3(92086248-92533253)x1** |
| **18** | **Hyperplasia** | **5q32q33.1(149546322-150948507)x3** |
| **19** | **SCC** | **3q26.32q28(178916549-189612296)x3** |
| **20** | **Hyperplasia** | **3p24.1q28(30648360-189612296)x3** |
| **22** | **SCC** | **3q26.32q28(178916549-189612296)x3** |
|  | **SCC** | **7q31.2(116339090-116436183)x4** |
| **24** | **Hyperplasia** | **19p13.2(8979173-9049628)x3** |
| **25** | **SCC** | **3q26.32q28(178916549-189612296)x3** |
|  | **SCC** | **7p11.2(55086931-55273315)x3** |
|  | **SCC** | **9p21.3(21968185-21994410)x1** |

SCC, squamous cell carcinoma; IN, intraepithelial neoplasia

**Supplementary Table 3B. Copy number alterations in background mucosa**

| **Case** | **Sample** | **iscn** |
| --- | --- | --- |
| **16** | **IN** | **3q26.32q28(178916549-189612296)x4** |
|  | **IN** | **6q25.2(152748821-152949490)x3** |
|  | **IN** | **8p23.1q24.23(11565791-139380232)x3** |

IN, intraepithelial neoplasia

**Supplementary Table 4. Copy number variants by real-time PCR**

|  | **CNV** | |  |  | **CNV** | |
| --- | --- | --- | --- | --- | --- | --- |
| **Case** | **TP53** | **NOTCH1** |  | **Case** | **TP53** | **NOTCH1** |
| **ESCC1 LVL** | **3** | **2** |  | **ESCC1 BM** | **2** | **2** |
| **ESCC2 LVL** | **2** | **2** |  | **ESCC2 BM** | **2** | **2** |
| **ESCC3 LVL** | **2** | **2** |  | **ESCC3 BM** | **2** | **2** |
| **ESCC4 LVL** | **1** | **2** |  | **ESCC4 BM** | **2** | **2** |
| **ESCC5 LVL** | **2** | **2** |  | **ESCC5 BM** | **2** | **2** |
| **ESCC6 LVL** | **2** | **2** |  | **ESCC6 BM** | **2** | **2** |
| **ESCC7 LVL** | **2** | **2** |  | **ESCC7 BM** | **2** | **2** |
| **ESCC8 LVL** | **2** | **2** |  | **ESCC8 BM** | **2** | **2** |
| **ESCC9 LVL** | **2** | **2** |  | **ESCC9 BM** | **2** | **2** |
| **ESCC10 LVL** | **2** | **2** |  | **ESCC10 BM** | **2** | **2** |
| **ESCC11 LVL** | **2** | **2** |  | **ESCC11 BM** | **2** | **2** |
| **ESCC12 LVL** | **2** | **2** |  | **ESCC12 BM** | **2** | **2** |
| **ESCC13 LVL** | **2** | **2** |  | **ESCC13 BM** | **2** | **2** |
| **ESCC14 LVL** | **2** | **3** |  | **ESCC14 BM** | **2** | **2** |
| **ESCC15 LVL** | **2** | **2** |  | **ESCC15 BM** | **2** | **2** |
| **ESCC16 LVL** | **2** | **2** |  | **ESCC16 BM** | **2** | **2** |
| **ESCC17 LVL** | **2** | **2** |  | **ESCC17 BM** | **2** | **2** |
| **ESCC18 LVL** | **2** | **2** |  | **ESCC18 BM** | **2** | **2** |
| **ESCC19 LVL** | **2** | **2** |  | **ESCC19 BM** | **2** | **2** |
| **ESCC20 LVL** | **2** | **2** |  | **ESCC20 BM** | **2** | **2** |
| **ESCC21 LVL** | **2** | **2** |  | **ESCC21 BM** | **2** | **2** |
| **ESCC22 LVL** | **1** | **1** |  | **ESCC22 BM** | **3** | **2** |
| **ESCC23 LVL** | **2** | **2** |  | **ESCC23 BM** | **3** | **2** |
| **ESCC24 LVL** | **3** | **2** |  | **ESCC24 BM** | **2** | **2** |
| **ESCC25 LVL** | **2** | **2** |  | **ESCC25 BM** | **2** | **2** |

ESCC, esophageal squamous cell carcinoma; LVL, Lugol-voiding lesion; BM, background mucosa

**Supplementary Table 5. Healthy control characteristics and mutational data**

| **Age, years (Mean ± SD)** | **74.2 ± 6.5** |
| --- | --- |
| **Gender (Male/Female)** | **2/3** |
| **Smoking habit (Yes/No)** | **1/4** |
| **Drinking habit (Yes/No)** | **2/3** |
|  |  |
| **Mutant genes, n** | **13** |
| **Somatic mutations, n** | **13** |
| **Coverage, median (range)** | **149 (18-274)** |
| **AF, % (range)** | **10 (4-28)** |
| **Missense, n (%)** | **12 (92)** |
| **Nonsense, n (%)** | **1 (8)** |

AF, allele frequency
